# High-resolution crystal structure of the Fv of quaternary neutralizing epitope mAb 2909 reveals atomic details of its antigen-binding site

**DOI:** 10.1186/1742-4690-9-S2-P79

**Published:** 2012-09-13

**Authors:** JM Sampson, A Killikelly, H Zhang, MK Gorny, S Zolla-Pazner, X Kong

**Affiliations:** 1 NYU School of Medicine, New York, NY, USA

## Background

Human mAb 2909 is in a class of potently neutralizing mAbs against the HIV-1 quaternary neutralizing epitope (QNE) preferentially presented by the native Env trimer complex. Its distinctive feature is a long CDR H3 loop with 2 sulfated tyrosines that are suggested to play a key role in antigen binding. Two structures of the Fab fragment of 2909 have been published, but at only 3.3Å and 3.2Å resolution, respectively, some atomic-level details of the antigen binding sites of these structures are contradictory.

## Methods

After crystallizing a recombinant Fv (rFv) of mAb 2909, expressed as a single chain in E. coli and refolded from inclusion bodies, we solved and refined its structure to 1.9Å resolution. We also characterized the neutralizing activity of the rFv against pseudotyped virus SF162.

## Results

Despite lacking the native sulfation of 2 tyrosine residues at the apex of CDR H3, rFv 2909 retains neutralization activity against SF162 pseudoviruses. Our high-resolution structure features a series of 5 tyrosine residues decorating one face of H3 like rungs of a spiral staircase, as seen in the Spurrier structure. The presence of this feature, despite different crystal packing around the H3 loop, suggests that the stacking pattern is not an artifact of crystallization, and that these tyrosine side chains play an important role in epitope recognition.

## Conclusion

Our structure of rFv 2909 at 1.9Å resolution reveals additional atomic-level details of its antigen-binding site, allowing further analysis of its binding mode. Our data demonstrate that rFv can be used as a tool to obtain high-resolution structures of antigen-binding regions, and may be useful for experiments requiring molecular weights smaller than that of a full Fab fragment, such as ITC and NMR spectroscopy.

